# Splenic artery aneurysm masquerading as an intraductal tubulopapillary neoplasm diagnosed by contrast-enhanced endoscopic ultrasound

**DOI:** 10.1055/a-2333-9361

**Published:** 2024-06-07

**Authors:** Takeshi Ogura, Kimi Bessho, Nobuhiro Hattori, Jun Matsuno, Hiroki Nishikawa

**Affiliations:** 1 13010 2nd Department of Internal Medicine, Osaka Medical and Pharmaceutical University, Osaka, Japan; 2 13010 Endoscopy Center, Osaka Medical and Pharmaceutical University, Osaka, Japan

The growth of intraductal tubulopapillary neoplasms is characterized by features such as filling, expansion, and proliferation of the main pancreatic duct (MPD) 1 . The resultant obstruction to the flow of pancreatic juice leads to MPD dilatation but, unlike with intraductal papillary mucinous neoplasms, cystic lesions and mucus are not observed. Splenic artery aneurysm, which is a rare condition, mainly occurs in the distal third of the splenic artery 2 . Although splenic artery aneurysms are usually asymptomatic 3 , rupture of the aneurysm can cause dramatic hypotensive shock, with a high mortality rate owing to intraperitoneal hemorrhage. Rarely, hemosuccus pancreaticus can occur because of rupture of a splenic artery aneurysm into the MPD 4 . In this condition, differentiating between an intraductal tubulopapillary neoplasm and a splenic artery aneurysm is not very challenging because splenic artery aneurysmal rupture is normally symptomatic; however, if a splenic artery aneurysm spontaneously ruptures into the MPD and immediately stops bleeding, the differential diagnosis can be challenging. We herein describe successful differentiation between these diseases using contrast-enhanced endoscopic ultrasound (EUS).

A 59-year-old man was admitted to our hospital owing to a pancreatic tumor. A computed tomography scan showed a stone in the pancreatic head, along with MPD dilatation ( Fig. 1 ). Magnetic resonance cholangiopancreatography showed a tumor-like lesion in the body of the MPD ( Fig. 2 ). EUS also showed an intraductal lesion, with thickening of the wall in the MPD ( Fig. 3 ). Based on these imaging findings, an intraductal tubulopapillary neoplasm was suspected. Because however EUS showed that the lesion was connected to the splenic artery ( Fig. 4 ), contrast-enhanced EUS was attempted. Contrast-enhanced EUS showed no vascularity within the tumor-like lesion, and, as a splenic artery aneurysm was observed, the tumor-like lesion was considered to be coagulum ( Video 1 ). Therefore, although this patient developed splenic artery aneurysm rupture into the MPD, there was fortunately spontaneous and immediate hemostasis. The patient subsequently underwent successful endovascular treatment, without any adverse events ( Fig. 5 ).

**Fig. 1:**
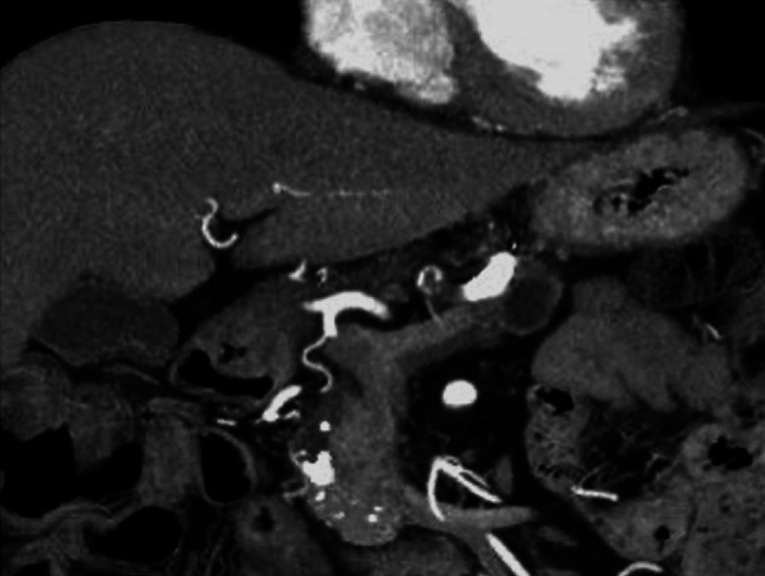
Computed tomography scan showing a stone in the pancreatic head, along with main pancreatic duct dilatation.

**Fig. 2:**
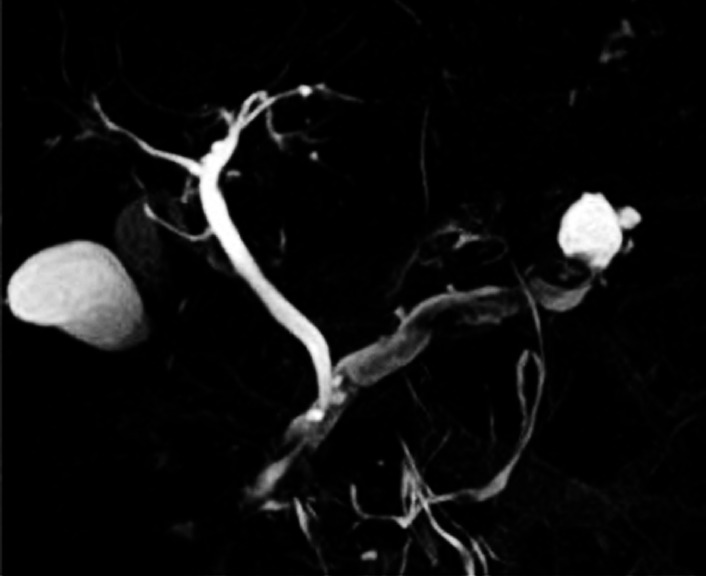
Magnetic resonance cholangiopancreatography showing a tumor-like lesion in the body of the main pancreatic duct.

**Fig. 3:**
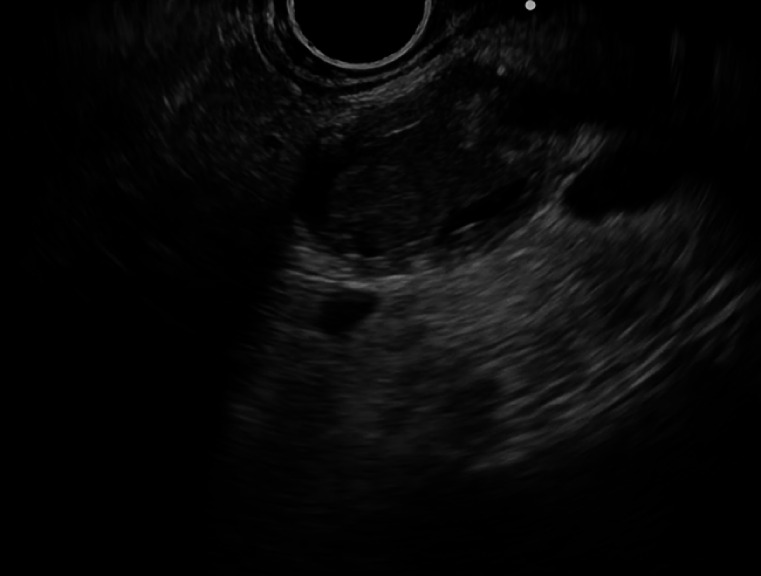
Endoscopic ultrasound image showing an intraductal lesion, with thickening of the wall in the main pancreatic duct.

**Fig. 4:**
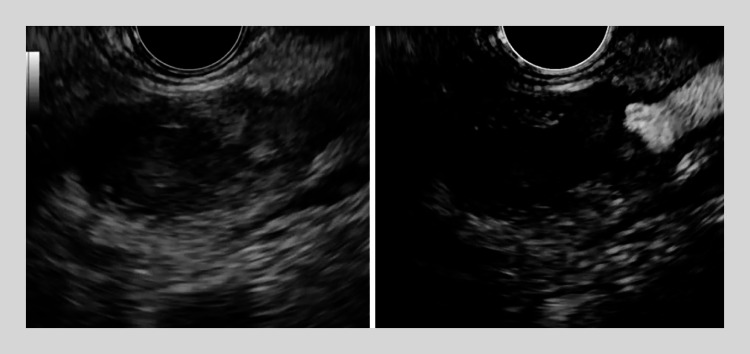
Contrast-enhanced endoscopic ultrasound images showing no vascularity within the tumor-like lesion and a splenic artery aneurysm, so the tumor-like lesion was considered to be coagulum.

A tumor-like lesion within the main pancreatic duct is shown on contrast-enhanced endoscopic ultrasound to be coagulum from a ruptured splenic artery aneurysm.Video 1

**Fig. 5:**
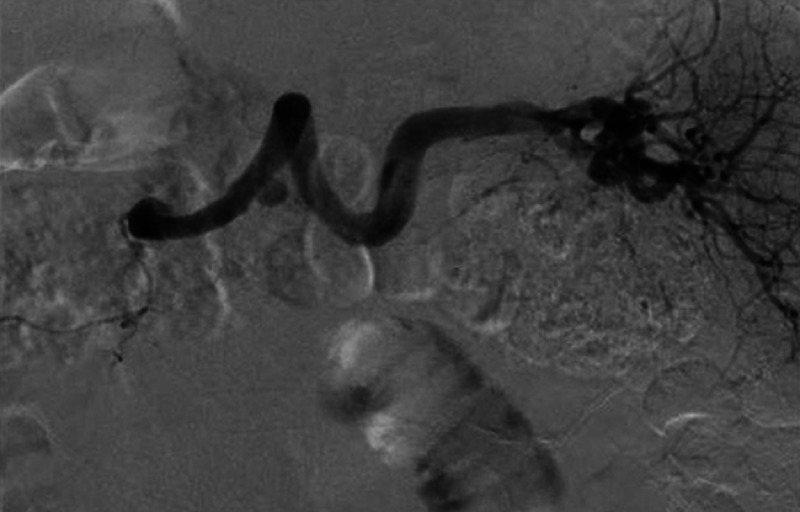
Fluoroscopic image prior to endovascular treatment of a splenic artery aneurysm.

In conclusion, pancreatic intraductal lesions should be carefully diagnosed, with due consideration given to rare conditions, such as splenic artery aneurysm rupture into the MPD, in the differential diagnosis.

Endoscopy_UCTN_Code_CCL_1AF_2AZ_3AD

## Notes

Endoscopy E-Videos https://eref.thieme.de/e-videos
*E-Videos* is an open access online section of the journal *Endoscopy* , reporting on interesting cases and new techniques in gastroenterological endoscopy. All papers include a high-quality video and are published with a Creative Commons CC-BY license. Endoscopy E-Videos qualify for HINARI discounts and waivers and eligibility is automatically checked during the submission process. We grant 100% waivers to articles whose corresponding authors are based in Group A countries and 50% waivers to those who are based in Group B countries as classified by Research4Life (see: https://www.research4life.org/access/eligibility/ ). This section has its own submission website at https://mc.manuscriptcentral.com/e-videos .
